# Effect of structured community-based older people education program on empathy, emotional intelligence, and caring behavior among nursing students

**DOI:** 10.3389/fmed.2025.1651669

**Published:** 2025-12-03

**Authors:** Jing Ma, PingLei Chui, Mei Chan Chong, Jingru Yuan, Yongyan Zhu, Luyao Liu, Zhenqing Sun

**Affiliations:** 1 Department of Nursing Science, Universiti Malaya Faculty of Medicine, Kuala Lumpur, Malaysia; 2 School of Nursing, Henan University of Science and Technology, Luoyang, China; 3 Henan Vocational College of Tuina, Luoyang, China

**Keywords:** community-based education, nursing students, empathy, emotional intelligence, caring behavior

## Abstract

**Background:**

The rapid aging of China’s population creates an urgent need for nursing students who possess core competencies in empathy, emotional intelligence, and caring behavior to meet the complex needs of older adults. Current educational approaches often emphasize technical skills at the expense of these essential humanistic qualities.

**Methods:**

This quasi experimental study evaluated the effectiveness of a 4 weeks Structured Community-based Older People Education (SCOPE) program, comparing it to standard curriculum. Participants included 190 third-year nursing students, assigned to either an intervention group (*n* = 96) receiving the SCOPE program or a control group (*n* = 94) receiving standard training. The SCOPE program combined aging simulations, clinical skills laboratories, standardized patient scenarios, and supervised community placements. Validated scales were used to measure outcomes at baseline (T0), immediately post-intervention (T1), and at an 8-week follow-up (T2). Data were analyzed using Generalized Estimating Equations.

**Results:**

Students in the SCOPE group showed significant improvements in total empathy (increasing from 81.25 ± 0.85 at T0 to 94.90 ± 1.24 at T2), emotional intelligence (from 122.39 ± 1.10 to 142.63 ± 1.31), and caring behavior (from 177.72 ± 1.60 to 204.77 ± 2.12). These gains were significantly greater than those observed in the control group at both T1 and T2 (*p* < 0.001). Analysis revealed sustained improvements across dimensions including cognitive empathy and emotional perception, while the control group demonstrated only minimal progress.

**Conclusion:**

SCOPE program significantly enhanced nursing students’ humanistic competencies with immediate and retained benefits, suggesting the value of integrating structured experiential learning into nursing curricula

## 1 Introduction

The global population over 60 is projected to reach 2.1 billion by 2050, with 80% residing in low- and middle-income countries (1). In China, this demographic transition is accelerated by declining fertility rates and increasing life expectancy, resulting in 264 million older adults (18.7% of the population) as of 2023 (2). This shift demands nurses equipped with technical proficiency and humanistic competencies—empathy, emotional intelligence (EI), and caring behavior—to address the multifaceted needs of older adults, including chronic disease management, cognitive decline, and social isolation.

Humanistic competencies in nursing are defined as integrating cognitive, emotional, and behavioral skills to deliver compassionate, patient-centered care. Empirical evidence identifies three pillars. Empathy is understanding and sharing patients’ emotional states while maintaining therapeutic boundaries (3). Empathetic nurses reduce elder mistreatment and enhance trust in care relationships (4, 5). Emotional Intelligence (EI) is the capacity to perceive, regulate, and utilize emotions to guide actions (6). High-EI nurses exhibit lower burnout rates and resolve conflicts more effectively (7, 8). Caring Behavior is the intentional acts demonstrating compassion and respect. Patients under the care of nurses with strong caring behaviors report higher satisfaction and adherence to treatment plans (9, 10). These competencies are not merely “soft skills” but critical determinants of care quality, particularly in geriatrics, where psychosocial needs often outweigh purely clinical demands.

Despite their significance, current nursing curricula disproportionately emphasize technical skills (e.g., wound care, medication administration) over humanistic development (11–13). Current nursing education in China faces key limitations, including insufficient integration of empathy and emotional regulation training in curricula, as evidenced by studies highlighting the need for structured interventions (14, 15). Additionally, pedagogical approaches remain heavily lecture-based, with limited opportunities for experiential learning, as demonstrated by critiques of traditional simulation debriefing methods (16). A survey among Chinese nursing students revealed that they felt unprepared to manage the psychosocial complexities of aging, citing insufficient training in communication and emotional regulation (17). This gap between education and real-world practice may leave nursing graduates unprepared to handle the emotional and ethical complexities of caring for older adults.

Delaying humanistic training until the clinical phase can hinder the development of professional identity and empathy in nursing students (18). Early exposure to humanistic values during education is key to fostering long-term compassionate practice (19). Without this foundation, nurses are more likely to adopt task-focused care in fast-paced clinical environments (20), and efforts to retrain empathy later often fail due to time pressure and stress in the workplace (21). Therefore, incorporating humanistic training during nursing education is crucial for maintaining core competencies and supporting long-term quality of care.

A survey of 7,774 older adults in Zhejiang Province (22) found that 52.1% were managing multiple chronic illnesses, highlighting the complexity of their physical and psychosocial needs (22). The tension between traditional filial expectations and the reality of urban migration adds further depth to caregiving interactions. Through engagement with this group, students gain experience in delivering holistic care, navigating cultural and generational dynamics, and challenging ageist assumptions by forming meaningful connections (23, 24). Such materials on education are irreplicable in hospital settings, where time constraints and acute care priorities limit relationship-building.

The initiative for improved humanistic competencies in nursing students has therefore encouraged various pedagogical approaches other than the traditional didactic method of teaching. There is increasing evidence supporting high-impact specific educational methods that could be used to develop such skills. First, experiential aging simulations whereby students wear devices that temporarily impair their ability to see or hear, and/or mobility has significantly enhanced empathy as well as attitudes toward older adults because it offers profound firsthand insight into daily challenges associated with aging (25–27). Second, standardized patient (SP) scenarios, which involve realistic clinical interactions with trained actors, have proven effective in developing students’ communication skills, emotional intelligence, and clinical reasoning in a safe and controlled environment, particularly in emotionally charged geriatric contexts (28–30). Finally, mentored community practicums offer the unique opportunity for students to apply theoretical knowledge in real-world settings, build therapeutic relationships with older adults over time, and understand the social determinants of health, leading to a more holistic and developmentally appropriate caring behavior (31, 32).

Though the isolated advantages of these techniques are well noted, there is no integrated educational program that systematically combines these three powerful modalities. This study aims to assess the effectiveness of the Structured Community-based Older People Education (SCOPE) program in enhancing nursing students’ empathy, emotional intelligence (EI), and caring behavior, compared to standard education. The SCOPE program utilizes a four-phase curriculum to address key gaps in nursing training: Experiential Aging Simulations, Skills Labs, Standardized Patient Scenarios, and Community Practicums. These phases integrate theory, simulation, and community engagement to foster empathy and EI, while promoting humanistic competencies are essential for high-quality geriatric care.

By evaluating the impact of the SCOPE program, this study aims to guide curriculum reforms that better align nursing education with the dynamic healthcare demands of aging populations. The findings will enhance nurses’ preparedness to deliver high-quality care for older adults, which is expected to improve health outcomes in this population.

## 2 Materials and methods

### 2.1 Study design

This non-randomized controlled trial employed a pre-test/post-test design. This study was conducted adhered by the Undergraduate Nursing Program Professional Accreditation Standards (33) and was reported following relevant elements of the Strengthening the Reporting of Observational studies in Epidemiology (STROBE) and Consolidated Standards of Reporting Trials (CONSORT) guidelines, adapted for quasi-experimental designs, to ensure transparency and methodological rigor (34–36). The Template for Intervention Description and Replication (TIDieR) checklist was utilized to detail the SCOPE program (e.g., session details, instructor qualifications) and control group activities (37, 38). The Guideline for Reporting Evidence-based practice Educational interventions and Teaching (GREET) checklist ensured comprehensive reporting of educational intervention components, including participant recruitment and outcome assessment procedures (39). Ethical approval was obtained from the university where the intervention was implemented (Approval No: 2023-0067). All participants provided written informed consent, and data were anonymized to protect confidentiality.

### 2.2 Study participants

A consecutive sample of 221 third-year undergraduate nursing students enrolled in community nursing courses at a university in China was recruited between February and September 2023. Participants were divided into two groups based on their course enrolment: those taking Community Nursing A were assigned to the intervention group receiving the Structured Community-Based Older People Education Program (SCOPE), while those taking Community Nursing B formed the control group receiving the Standard Community-Based Older People Education Program. Eligibility screening was conducted according to predefined inclusion and exclusion criteria. Inclusion criteria were Enrolment in Semester 5–6 (year 3) of the Bachelor of Science in Nursing (BSN) program; completion of prerequisite courses: Fundamentals of Nursing I-II (Semesters 2–3); Health Assessment Practice (Semester 3); Medical/Surgical Nursing I (Semester 4); Active registration in Community Nursing A (intervention group) or B (control group); Provision of written informed consent. Exclusion criteria were: Documented mental health conditions or communication impairments affecting community practice; Academic suspension or withdrawal during the study period; and refusal to provide participation consent.

### 2.3 Intervention protocol

#### 2.3.1 SCOPE program intervention protocol

**FIGURE 1:**
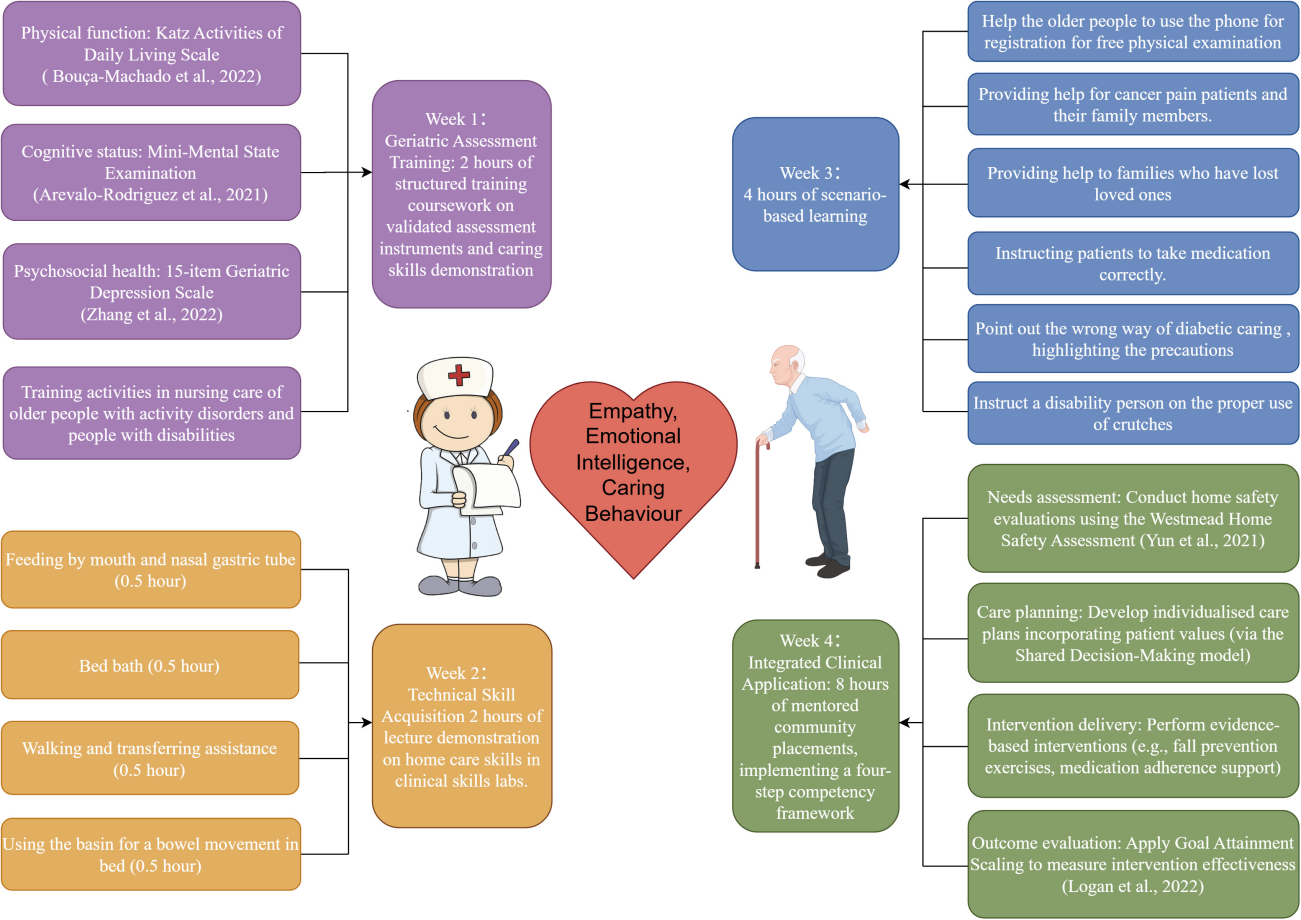
Weekly modules of SCOPE program targeting core competencies in geriatric care.

The Structured Community-based Older People Education (SCOPE) program was implemented for 4 weeks, with weekly modules targeting core competencies in geriatric care (Figure 1). The SCOPE program’s 4-week curriculum was precisely designed to provide an integrated approach to developing humanistic competencies. Each week integrated specific pedagogical strategies directly targeting empathy, emotional intelligence (EI), and caring behaviors through a combination of theoretical instruction, simulation, and real-world application. The design and content of each module were rigorously developed by the expert panel (*n* = 8), including geriatric nurses, nursing educators, and clinical psychologists, and aligned with the National Competency Standards for Nursing Education (40). Training sessions incorporated case-based simulations where students interpreted assessment results for standardized patients, followed by guided reflective discussions to enhance empathy through perspective-taking (41). Scenario-based learning was facilitated through standardized patient encounters, with 360° feedback from patients, caregivers, and nurse mentors to refine caring behaviors (42). Certified community nurse preceptors conducted live demonstrations using high-fidelity geriatric patient simulators, with immediate video-assisted debriefing to reinforce emotional regulation strategies (43).

During Week 1, students engage with validated geriatric assessment instruments (e.g., Katz Activities of Daily Living Scale, Mini-Mental State Examination, Geriatric Depression Scale), followed by structured demonstrations of caring skills. This phase does not only impart technical competence in conducting assessments, but through guided reflection on patients’ functional, cognitive, and psychosocial limitations, encourages perspective taking and emotional resonance, thereby fostering empathy. In Week 2, the achievement of clinical skills such as feeding assistance, bed bathing, walking and transfer support, or crutch instruction is conducted through hands-on demonstrations. These tasks are paired with reflective feedback on communication style, emotion regulation, and relational sensitivity during direct care, which explicitly targets the enhancement of emotional intelligence, particularly in recognizing and responding to patients’ emotional cues. Week 3 introduces scenario based learning, in which learners confront complex and emotionally charged caregiving situations (e.g., supporting bereaved families, guiding medication adherence, or addressing cancer pain). By requiring students to manage their own emotional reactions while demonstrating professional compassion, this stage consolidates both emotional intelligence and caring behavior in dynamic interactions. Finally, Week 4 situates trainees in mentored community placements, where they develop individualized care plans through shared decision making, conduct home safety assessments, and implement evidence based interventions (e.g., fall prevention exercises, adherence support). These integrative activities not only solidify clinical competence but also demand sustained commitment to caring behavior as observable practice, ensuring that empathy and emotional intelligence are continuously translated into patient centered actions.

Intervention fidelity was ensured through multiple strategies. First, all community nurse mentors completed a 16-h preceptor certification program, with high inter-rater reliability for skill competency evaluations (κ = 0.82). Second, protocol standardization was supported by a video-recorded demonstration library, accessible via the institutional learning management system. Third, quality monitoring was conducted through weekly audits of 20% of randomly selected sessions using the SCOPE Adherence Checklist, demonstrating strong internal consistency (Cronbach’s α = 0.91).

#### 2.3.2 The standard community-based older people education program

The control group participated in a 16-h Standard Community-based Education program delivered for 4 weeks (4 h/week), comprising routine clinical training at community clinics, activity centers, and residents’ homes. Activities included practical skill development sessions (e.g., health education delivered as a group meeting that included the older people in communities, physiotherapy and chronic disease management under nurse supervision, observational learning in public health education, and daily care provision for older adults). Students were required to submit two self-reported case analyses documenting their application of emotional and professional evoke moments during interactions; however, these reflections were unstructured and lacked guided competency frameworks. Unlike the intervention group’s systematic SCOPE program, the standard curriculum emphasized task-based procedural competencies without dedicated modules for emotional skill cultivation or mentored scenario simulations. This conventional approach served as the baseline comparator for evaluating the enhanced training outcomes.

#### 2.3.3 Follow-up

The data assessors were trained. Each participant received a face-to-face assessment and interview to minimize bias. All outcome procedures were performed at each time point: baseline (T0), 4 weeks post-intervention (T1), and 8 weeks post-intervention.

### 2.4 Instruments

Data were collected using a self-administered questionnaire comprising four sections: demographic characteristics (14 items), the 22-item Chinese College Student’s Empathy Questionnaire (CCSEQ; cognitive, emotional, and behavioral empathy dimensions; Cronbach’s α = 0.881) (44), the 33-item Chinese Emotional Intelligence Scale (EIS; four dimensions including emotional perception and regulation; Cronbach’s α = 0.897) (45), and the 37-item Chinese Caring Ability Inventory (CAI; three dimensions: acknowledge, patience, and courage; Cronbach’s α = 0.815) (46). All scales utilized Likert-type responses (5- or 7-point) and were grounded in Watson’s Theory of Human Caring (47). A pilot study (*n* = 30) confirmed robust psychometric properties: test-retest reliability over 1 month yielded intraclass correlation coefficients (ICC) of 0.82 (CCSEQ), 0.85 (EIS), and 0.79 (CAI). Exploratory factor analysis (EFA) demonstrated strong construct validity, with Kaiser-Meyer-Olkin (KMO) measures ≥ 0.846 and significant Bartlett’s tests (*p* < 0.001) for all scales. Varimax-rotated factor loadings ranged from 0.385 to 0.829, explaining 67.8–72.4% of total variance across scales. Internal consistency for subscales exceeded 0.70, except for self-emotion regulation (α = 0.595). Normality assessments revealed skewed distributions, necessitating non-parametric analyses. Pre-post pilot comparisons using Wilcoxon tests indicated significant improvements in cognitive empathy (*p* < 0.05) and caring behavior dimensions (*p* < 0.05), validating the instruments’ sensitivity to detect intervention effects.

### 2.5 Statistical analysis

Data were analyzed using SPSS 27.0 (IBM Corp.) with a two-tailed significance threshold of α = 0.05. Continuous variables approximating normality (Shapiro-Wilk test, *p* > 0.05) were expressed as mean ± SD, while categorical variables were summarized as frequencies and percentages. Group comparisons utilized independent *t*-tests for normally distributed scale scores. Chi-square/Fisher’s exact tests for demographic comparisons (gender, single-child status, residence type, etc.). Longitudinal changes in empathy, emotional intelligence, and caring behavior across baseline (T0), post-intervention (T1), and follow-up (T2) were assessed via Generalized Estimating Equations (GEE). The model incorporated baseline scores as covariates to control for initial imbalances and included three predictors: Group (intervention/control), Time (T0/T1/T2), and Group*Time interaction. The Least Squares (LS) estimates reflect the GEE-derived adjusted group differences, grounded in the Generalized Least Squares (GLS) theory extended by Vanegas et al. (48). By modeling the mean-variance relationship of the response variable, these estimates effectively isolate true intervention effects from correlated data variability. A stratified gate-keeping approach was implemented. Interaction effects were prioritized, with nonsignificant terms removed to test main effects. Robust standard errors accounted for within-subject correlations in repeated measures. Identity link function and exchangeable correlation structure were specified, given approximately normal residuals. Effect sizes were reported with 95% confidence intervals. All analyses adhered to intention-to-treat principles, with missing data handled via multiple imputations (five iterations, predictive mean matching). Sensitivity analyses confirmed model stability across complete-case and per-protocol cohorts.

## 3 Results

**FIGURE 2:**
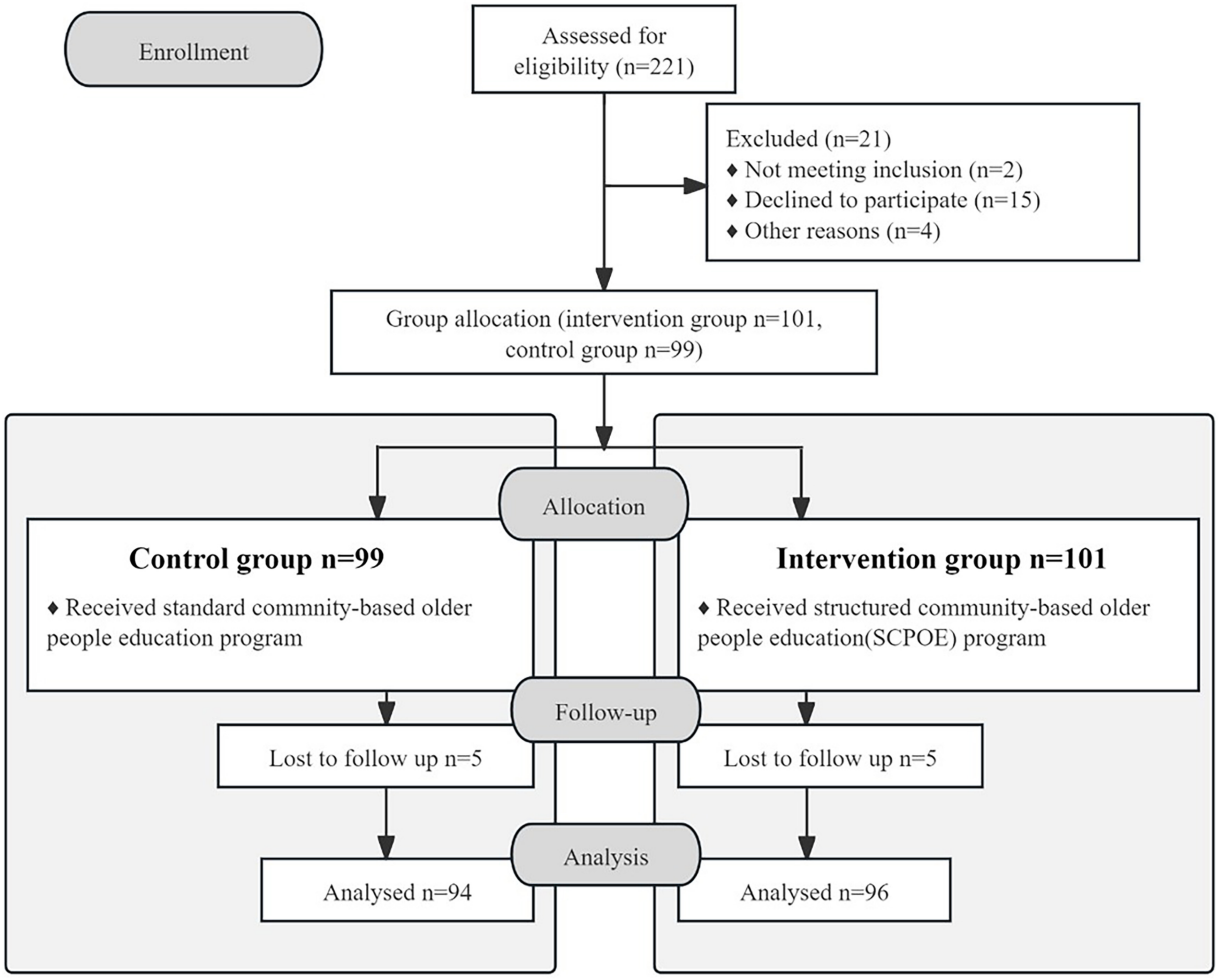
The research framework of this study.

A total of 221 students were registered in the third year of the nursing baccalaureate program, with 21 students excluded. Among this total, 2 students did not meet the inclusion criteria, 15 declined to participate, and 4 were excluded due to other reasons, such as scheduling conflicts and lack of availability during the study period. A total of 101 students were allocated to the intervention group and 99 were allocated to the control group. During the follow-up phase, 10 participants (5 from each group) were lost to follow-up. Reasons for loss to follow-up included personal factors. Ultimately, the intervention group provided 96 valid responses, achieving a completion rate of 95%, while the control group yielded 94 valid responses, with a completion rate of 94.9% (Figure 2). These figures were achieved after excluding incomplete responses and responses with completion times under 360 seconds, as these were considered invalid.

### 3.1 Participant characteristics

The intervention (*N* = 96) and control (*N* = 94) groups demonstrated comparable baseline profiles (Table 1). Participants were predominantly female (67.9%), not the single child (59.5%), and born in the same Province (87.9%). Over 93% had parents with high school or lower education, 50.5% voluntarily chose nursing majors, and 64.2% reported prior community service experience. No significant between-group differences were observed in gender distribution, voluntary major selection, cohabitation with older/disabled individuals, or regional origin (all *p* ≥ 0.05), confirming baseline equivalence for outcome attribution.

**TABLE 1:** The demographic characteristics of participants in the intervention (*N* = 96) and control group (*N* = 94).

Baseline characteristic		Intervention		Control		Total		χ^2^	*P*
		n	%	n	%	n	%		
Gender	Male	35	36.46	26	27.66	61	32.11	1.687	0.194
	Female	61	63.54	68	72.34	129	67.89		
Single child	Yes	37	38.54	40	42.55	77	40.53	0.317	0.573
	No	59	61.46	54	57.45	113	59.47		
Place of birth	Within province	86	89.58	81	86.17	167	87.89	0.52	0.471
	Outside province	10	10.42	13	13.83	23	12.11		
Father’s education level	High school and below	90	93.75	87	92.55	177	93.16	0.107	0.744
	Undergraduate and higher degrees	6	6.25	7	7.45	13	6.84		
Mother’s education level	High school and below	93	96.88	91	96.81	184	96.84	<0.001	1.000
	Undergraduate and higher degrees	3	3.13	3	3.19	6	3.16		
Student leader	Yes	58	60.42	58	61.70	116	61.05	0.033	0.856
	No	38	39.58	36	38.30	74	38.95		
Voluntarily take nursing as a major	Yes	51	53.13	45	47.87	96	50.53	0.524	0.469
	No	45	46.88	49	52.13	94	49.47		
Living with old people	Yes	63	65.63	56	59.57	119	62.63	0.743	0.389
	None	33	34.38	38	40.43	71	37.37		
Community-service experience	Yes	63	65.63	59	62.77	122	64.21	0.169	0.681
	None	33	34.38	35	37.23	68	35.79		

### 3.2. Primary outcomes: the effect of the SCOPE program on empathy, emotional intelligence and caring behavior: comparison between the intervention and control group at T0, T1, and T2

The SCOPE program led to significant and sustained improvements in empathy, emotional intelligence, and caring behavior among nursing students compared to standard training. According to Table 2, the total empathy score (CCSEQ) in the intervention group increased markedly from T0 (81.25 ± 0.85) to T1 (86.92 ± 0.84), and further to T2 (94.90 ± 1.24), while the control group showed a decline at T1 (74.24 ± 1.06) followed by partial recovery at T2 (87.39 ± 1.48). Between-group comparisons indicated significantly higher empathy scores in the intervention group at both T1 and T2 (*p* < 0.001). All three empathy subdimensions demonstrated similar trends: cognitive empathy increased from 22.91 to 26.23, emotional empathy from 30.15 to 34.55, and behavioral empathy from 29.70 to 34.11 in the intervention group. These improvements were statistically significant within groups over time (*p* < 0.001), and between-group comparisons at T1 and T2 also reached significance in most dimensions.

**TABLE 2:** Comparison of the effects of the SCOPE program (intervention group, *N* = 96) and the standard program (control group, *N* = 94) on total CCSEQ scores.

Item		Baseline (T0) Mean (SD)	Post-intervention (T1) Mean (SD)	Follow-up (T2) Mean (SD)	Wald chi-square	*P*
Cognitive empathy dimension	Intervention group	22.91 (0.29)	23.92 (0.28)	26.23 (0.37)	67.940	<0.001
	Control group	22.20 (0.25)	20.40 (0.33)	24.07 (0.42)	53.744	<0.001
	LS mean (95%CI)	0.70(−0.04, 1.45)	3.51***(2.66, 4.37)	2.15***(1.06, 3.25)		
	Wald chi-square	3.412	64.814	14.949		
	*P*	0.065	<0.001	<0.001		
Emotional empathy dimension	Intervention group	30.15 (0.34)	31.75 (0.35)	34.55 (0.54)	79.227	<0.001
	Control group	29.30 (0.342)	31.78 (0.551)	31.78 (0.551)	63.290	<0.001
	LS mean (95%CI)	0.85(−0.09, 1.79)	4.78***(3.73, 5.83)	2.78***(1.26, 4.29)		
	Wald chi-square	3.126	79.847	12.905		
	*P*	0.077	<0.001	<0.001		
Behavioral empathy dimension	Intervention group	29.70 (0.30)	31.25 (0.33)	34.11 (0.54)	94.410	<0.001
	Control group	28.99 (0.35)	26.87 (0.42)	31.54 (0.55)	55.476	<0.001
	LS mean (95%CI)	0.709(−0.190, 1.607)	4.38***(3.34, 5.42)	2.57***(1.05, 4.09)		
	Wald chi-square	2.388	68.138	11.018		
	*P*	0.122	<0.001	<0.001		
Empathy total score	Intervention group	81.25 (0.85)	86.92 (0.84)	94.90 (1.24)	121.098	<0.001
	Control group	80.49 (0.81)	74.24 (1.06)	87.39 (1.48)	67.546	<0.001
	LS mean (95%CI)	2.26(−0.05, 4.57)	12.67***(10.02, 15.33)	7.50***(3.71, 11.29)		
	Wald chi-square	3.678	87.537	15.063		
	*P*	0.055	<0.001	<0.001		

*p < 0.05, **p < 0.01,

***p < 0.001 (two-tailed tests; significance symbols refer to the LS mean).

Emotional intelligence, as shown in Table 3, also significantly improved in the intervention group. The total EIS score rose from 122.39 ± 1.10 at T0 to 142.63 ± 1.31 at T2, while the control group’s increase was smaller, from 122.80 ± 1.16 to 129.29 ± 1.62. Significant between-group differences were found at both T1 and T2 (*p* < 0.001). Among EIS subdimensions, the Emotional Perception score improved notably from 41.61 to 51.66, with the intervention group significantly outperforming controls at T2 (*p* < 0.001). Self-emotion Regulation increased from 30.13 to 33.96 (*p* < 0.001), and the Understanding Others’ Emotions dimension rose from 23.03 to 26.21, while the control group showed no significant change in this dimension (*p* = 0.128). Additionally, the Use of Emotion increased from 27.61 to 30.80, a statistically significant change compared with the control group (*p* < 0.001).

**TABLE 3:** Comparison of the effects of the SCOPE program (intervention group, *N* = 96) and the standard program (control group, *N* = 94) on total EIS scores.

Item		Baseline (T0) Mean (SD)	Post-intervention (T1) Mean (SD)	Follow-up (T2) Mean (SD)	Wald chi-square	*P*
Emotional perception dimension	Intervention	41.61 (0.47)	47.39 (0.71)	51.66 (0.73)	204.601	<0.001
	Control	41.98 (0.45)	42.99 (0.60)	45.61 (0.73)	17.242	<0.001
	LS mean (95%CI)	−0.36(−1.63, 0.91)	4.40***(2.58, 6.21)	6.05***(4.02, 8.08)		
	Wald chi-square	0.316	22.522	34.245		
	*P*	0.574	<0.001	<0.001		
Self-emotion regulation dimension	Intervention	30.13 (0.30)	32.75 (0.36)	33.96 (0.38)	74.948	<0.001
	Control	30.61 (0.30)	30.41 (0.38)	31.84 (0.47)	11.476	0.003
	LS mean (95%CI)	−0.48(−1.32, 0.35)	2.34***(1.30, 3.37)	2.12***(0.93, 3.30)		
	Wald chi-square	1.273	19.613	12.246		
	*P*	0.259	<0.001	<0.001		
Understanding others’ emotion dimension	Intervention	23.03 (0.25)	24.79 (0.30)	26.21 (0.31)	92.437	<0.001
	Control	22.39 (0.28)	22.63 (0.33)	23.27 (0.39)	4.112	0.128
	LS mean (95%CI)	0.64(−0.09, 1.37)	2.16***(1.30, 3.03)	2.94***(1.96, 3.92)		
	Wald chi-square	2.910	24.075	34.737		
	*P*	0.088	<0.001	<0.001		
Use emotion dimension	Intervention	27.61 (0.29)	29.58 (0.32)	30.80 (0.31)	76.115	<0.001
	Control	27.82 (0.33)	27.57 (0.36)	28.57 (0.40)	4.907	0.086
	LS mean (95%CI)	−0.20(−1.07, 0.67)	2.01***(1.08, 2.94)	2.23***(1.24, 3.22)
	Wald chi-square	0.212	17.828	19.445
	*P*	0.645	<0.001	<0.001
EIS total score	Intervention	122.39 (1.10)	134.51 (1.53)	142.63 (1.31)	231.016	<0.001
	Control	122.80 (1.16)	123.61 (1.46)	129.29 (1.62)	15.791	<0.001
	LS mean (95%CI)	−0.41(−3.55, 2.72)	10.90***(6.76, 15.05)	13.34***(9.26, 17.42)		
	Wald chi-square	0.066	26.585	41.039		
	*P*	0.797	<0.001	<0.001		

*p < 0.05, **p < 0.01, ***p < 0.001 (two-tailed tests; significance symbols refer to the LS mean).

Similarly, the CAI total score (Table 4) in the intervention group increased from 177.72 ± 1.60 at baseline to 204.77 ± 2.12 at T2, compared to the control group, which rose more modestly from 173.79 ± 1.42 to 191.22 ± 2.45. Significant between-group differences emerged at T1 (*p* = 0.024) and were even more pronounced at T2 (*p* < 0.001). Within the caring behavior subdimensions, the Patience score showed the most substantial increase (from 48.20 to 67.66, *p* < 0.001), followed by Acknowledge (from 72.39 to 77.33, *p* < 0.001). While Courage also increased (from 57.14 to 59.78), its early changes were less significant, particularly at T1 (*p* = 0.030), but became more apparent by T2 (*p* < 0.001). These multidimensional improvements across empathy, emotional intelligence, and caring behaviors provide strong evidence of the intervention’s effectiveness in enhancing nursing students’ professional competencies.

**TABLE 4:** Comparison of the effects of the SCOPE program (intervention group, *N* = 96) and the Standard program (Control Group, *N* = 94) on total CAI score.

Item		Baseline (T0) Mean (SD)	Post-intervention (T1) Mean (SD)	Follow-up (T2) Mean (SD)	Wald chi-square	*P*
CAI acknowledge dimension	Intervention	72.39 (0.81)	74.88 (1.04)	77.33 (1.00)	29.842	<0.001
	Control	71.29 (0.88)	72.88 (0.93)	73.77 (1.13)	4.133	0.127
	LS mean (95%CI)	1.10(−1.25, 3.45)	1.99(−0.73, 4.71)	3.57**(0.65, 6.48)		
	Wald chi-square	0.838	2.056	5.751		
	*P*	0.360	0.152	0.016		
CAI patience dimension	Intervention	48.20 (0.87)	60.84 (1.12)	67.66 (1.24)	130.193	<0.001
	Control	46.38 (0.86)	57.82 (1.31)	61.32 (1.14)	113.538	<0.001
	LS mean (95%CI)	1.81(−0.58, 4.21)	3.02(−0.34, 6.39)	6.34(3.04, 9.64)***		
	Wald chi-square	2.206	3.096	14.160		
	*P*	0.137	0.078	<0.001		
CAI courage dimension	Intervention	57.14 (0.62)	57.69 (0.64)	59.78 (0.62)	14.085	<0.001
	Control	56.12 (0.58)	55.67 (0.68)	56.14 (0.76)	0.576	0.750
	LS mean (95%CI)	1.02(−0.64, 2.68)	2.02**(0.19, 3.84)	3.64***(1.72, 5.57)		
	Wald chi-square	1.451	4.701	13.748		
	*P*	0.228	0.030	<0.001		
CAI total score	Intervention	177.72 (1.60)	193.41 (2.20)	204.77 (2.12)	138.947	<0.001
	Control	173.79 (1.42)	186.37 (2.22)	191.22 (2.45)	54.316	<0.001
	LS mean (95%CI)	3.93(−0.26, 8.12)	7.03**(0.91, 13.16)	13.55***(7.19, 19.91)		
	Wald chi-square	3.377	5.063	17.420		
	*P*	0.066	0.024	<0.001		

*p < 0.05,

**p < 0.01,

***p < 0.001 (two-tailed tests; significance symbols refer to the LS mean).

### 3.3 Secondary outcomes: GEE model results for the intervention group

To examine the specific effect of the SCOPE program over time, GEE analyses were conducted for the intervention group (Tables 5–7). In empathy (Table 5), significant increases were observed from T1 to T3 (β = 9.55, 95% CI: 7.59–11.51, *p* < 0.001), with an even larger jump from T1 to T3 in the total score (Group T3: *β* = 12.15, *p* < 0.001). Emotional and behavioral empathy dimensions followed a similar trend, with T3T1 changes exceeding 3.4 points in both dimensions (*p* < 0.001). Emotional intelligence (Table 6) also showed a strong and consistent increase over time. The EIS total score rose by 13.44 points from T0 to T2 (β = 13.44, 95% CI: 10.95–15.92), with a group-time interaction effect (Group*t2) reaching β = 20.24 (*p* < 0.001). Subdimensions such as self-emotion regulation and understanding others’ emotions also showed robust growth, with β coefficients of 3.83 and 3.18, respectively (*p* < 0.001). Similarly, for caring behavior (Table 7), the intervention group exhibited a marked increase in CAI total score from T0 to T2 (β = 22.29, *p* < 0.001), and group-by-time interaction at T2 yielded a large effect size (β = 27.05, 95% CI: 22.42–31.69, *p* < 0.001). Across dimensions (acknowledge, patience, courage), most showed statistically significant and sustained improvement, confirming the long-term efficacy of the program.

**TABLE 5:** GEE model regression coefficients and odds ratios in CCSEQ.

	LS mean (95% CI) (mean difference)	β (regression coefficient, parameter estimation)	OR(95%CI)	Wald chi-square	*P*
**Cognitive empathy dimension**					
Time*Time					
T2*T1	−0.38 (−0.92, 0.16)	1.010**(0.316, 1.705)	2.747 (1.372, 5.501)	8.132	0.004
T3*T1	2.61(2.02, 3.19)	3.323***(2.530, 4.116)	27.741 (12.551, 61.341)	67.437	<0.001
T3*T2	2.98(2.31, 3.66)				
Group*Time					
Group*T3	3.32(2.53, 4.12)	3.323***(2.530, 4.116)	27.741(12.551, 61.314)	67.437	<0.001
Group*T2	1.01(0.32, 1.70)	1.010**(0.316, 1.705)	2.747(1.372, 5.501)	8.132	0.004
**Emotional empathy dimension**					
Time*Time					
T2*T1	−0.34(−1.05, 0.37)	−0.342(0.365, 0.898)	0.710(0.350, 1.441)	0.898	0.343
T3*T1	3.45(2.63, 4.247)	3.453***(2.630, 4.275)	31.583(13.880, 71.869)	67.737	<0.001
T3*T2	3.79(2.87, 4.71)				
Group*Time					
Group*T3	4.41(3.37, 5.45)	4.406***(3.367, 5.446)	81.962(28.991, 231.715)	69.054	<0.001
Group*T2	1.60(0.72, 2.49)	1.604***(0.717, 2.491)	4.974(2.049, 12.074)	12.568	<0.001
**Behavioral empathy dimension**					
Time*Time					
T2*T1	−0.26(−0.94, 0.41)	−0.263(−0.935, 0.409)	0.769 (0.392, 505)	0.589	0.443
T3*T1	3.49(2.70, 4.29)	3.495***(2.701, 4.289)	32.492 (14.892, 72.869)	74.432	<0.001
T3*T2	3.76 (2.87, 4.64)				
Group*Time					
Group*T3	4.42(3.46, 5.38)	4.417***(5.375, 81.519)	82.820(31.750, 216.034)	81.519	<0.001
Group*T2	1.55(0.77, 2.33)	1.552***(0.771, 2.333)	4.721(2.162, 10.311)	15.167	<0.001
**CCSEQ total score**					
Time*Time					
T2*T1	−0.98(−2.69, 0.73)	−0.984(−2.695, 0.726)	0.374(0.068, 2.067)	1.272	0.259
T3*T1	9.55(7.59, 11.51)	9.553***(7.591, 11.514)	14081.703(1979.996, 100148.845)	91.084	<0.001
T3*T2	10.54(8.30, 12.77)				
Group*Time					
Group*T3	12.15 (9.89, 14.40)	12.146***(9.892, 14.399)	188307.837 (19776.770, 1793004.701)	111.586	<0.001
Group*T2	4.17 (2.08, 6.25)	4.167***(2.080, 6.254)	64.50 (8.002, 519.912)	15.312	<0.001

*p < 0.05,

**p < 0.01,

***p < 0.001 (two-tailed tests; significance symbols refer to the β coefficients).

**TABLE 6:** GEE model regression coefficients and odds ratios in EI.

	LS mean (95% CI)	β	OR(95%CI)	Wald chi-square	*P*
**Emotional perception dimension**					
Time*Time					
T1*T0	3.42(2.36, 4.48)	3.416***(2.356, 4.476)	30.441(10.545, 87.878)	39.878	<0.001
T2*T0	6.87(5.56, 8.08)	6.868***(5.654, 8.083)	961.429(285.329, 3239.580)	122.805	<0.001
T2*T1	3.45(2.32, 4.59)				
Group*Time					
Group*T2	10.04(8.63, 11.46)	10.042***(8.626, 11.457)	22963.624(5574.906, 94589.574)	193.288	<0.001
Group*T1	5.77(4.32, 7.22)	5.771***(4.323, 7.218)	320.805(75.437, 1364.263)	61.054	<0.001
**Self-emotion regulation dimension**					
Time*Time					
T1*T0	1.23(0.59, 1.87)	1.232***(0.593, 1.870)	3.427(1.809, 6.491)	14.275	<0.001
T2*T0	2.55(1.87, 3.23)	2.547***(1.868, 3.227)	12.773(6.476, 25.193)	54.037	<0.001
T2*T1	1.32(0.72, 1.91)				
Group*Time					
Group*T2	3.83(2.95, 4.72)	3.833***(2.947, 4.719)	46.216(19.055, 112.095)	71.907	<0.001
Group*T1	2.63(1.77, 3.48)	1.766***(3.484, 35.908)	13.805(5.850, 32.576)	35.908	<0.001
**Understanding others’ emotion dimension**					
Time*Time					
T1*T0	1.01(0.50, 1.51)	1.005***(0.501, 1.510)	2.733(1.650, 4.526)	15.245	<0.001
T2*T0	2.04(1.48, 2.60)	2.037***(1.476, 2.598)	7.666(4.375, 13.434)	50.653	<0.001
T2*T1	1.03(0.49, 1.57)				
Group*Time					
Group*T2	3.18(2.53, 3.83)	3.177***(2.526, 3.828)	23.977(12.499, 45.993)	91.381	<0.001
Group*T1	1.76(1.06, 2.46)	1.760***(1.065, 2.456)	5.815(2.900, 11.661)	24.590	<0.001
**Use of emotion dimension**					
Time*Time					
T1*T0	0.87(0.31, 1.43)	0.874**(0.313, 1.435)	2.396(1.367, 4.199)	9.313	0.002
T2*T0	1.98(1.36, 2.61)	1.984***(1.360, 2.609)	7.273(3.895, 13.581)	38.781	<0.001
T2*T1	1.11(0.51, 1.71)				
Group*Time					
Group*T2	3.19(2.44, 3.94)	3.188***(2.438, 3.937)	24.228(11.454, 51.249)	69.534	<0.001
Group*T1	1.97(1.27, 2.67)	1.969***(1.267, 2.671)	7.162(3.549, 14.453)	30.198	<0.001
**EIS total score**					
Time*Time					
T1*T0	6.53(4.09, 8.96)	6.526***(4.094, 8.959)	682.878(59.963, 7776.824)	27.650	<0.001
T2*T0	13.44(10.95, 15.92)	13.437***(10.951, 15.923)	684772.610(57016.027, 8224240.618)	112.247	<0.001
T2*T1	6.91(4.69, 9.13)				
Group*Time					
Group*T2	20.24(17.62, 22.86)	20.240***(17.623, 22.856)	616508910.2(45029661.51, 8440730478)	229.813	<0.001
Group*T1	12.13(8.89, 15.36)	12.125***(8.890, 15.360)	184425.34(7259.254, 4685427.277)	53.966	<0.001

*p < 0.05,

**p < 0.01,

***p < 0.001 (two-tailed tests; significance symbols refer to the β coefficients).

**TABLE 7:** GEE model regression coefficients and odds ratios in CAI.

	LS mean (95% CI)	β	OR(95%CI)	Wald chi-square	*P*
**CAI acknowledge dimension**					
Time*Time					
T1*T0	2.05 (0.49, 3.61)	2.047** (0.490, 3.605)	7.747 (1.632, 36.787)	6.636	0.010
T2*T0	3.73 (2.22, 5.23)	3.726*** (2.224, 5.229)	41.526 (9.240, 186.617)	23.620	<0.001
T2*T1	1.68 (0.14, 3.22)				
Group*Time					
Group*T2	4.95(3.15, 6.75)	4.948**(3.145, 6.750)	140.881(23.229, 854.423)	28.946	<0.001
Group*T1	2.49(0.45, 4.53)	2.490**(0.446, 4.533)	12.056(1.562, 93.080)	5.700	0.017
**CAI patience dimension**					
Time*Time					
T1*T0	12.05 (9.93, 14.17)	12.047*** (9.926, 14.169)	170649.738 (20454.311, 1423725.914)	123.887	<0.001
T2*T0	17.22 (14.96, 19.48)	17.221*** (14.958, 19.484)	30130624.31 (3134234.995, 289657451.7)	222.426	<0.001
T2*T1	5.17 (3.01, 7.34)				
Group*Time					
Group*T2	19.46(16.05,22.87)	19.458*** (16.046, 22.871)	282258346.9 (9300555.214, 8566131007)	124.882	<0.001
Group*T1	12.65(9.67, 15.62)	12.646**(9.673, 15.619)	310467.136 (15881.321, 6069384.468)	69.506	<0.001
**CAI courage dimension**					
Time*Time					
T1*T0	0.06 (−0.93, 1.05)	0.058 (−0.934, 1.050)	1.060 (0.393, 2.857)	0.013	0.909
T2*T0	1.35 (0.31, 2.38)	1.347**(0.31, 22.383)	3.847 (1.366, 10.840)	6.500	0.011
T2*T1	1.29 (0.26, 2.32)				
Group*Time					
Group*T2	2.65(1.26, 4.03)	2.646**(1.257, 4.034)	14.095(3.516, 56.511)	13.947	<0.001
Group*T1	0.55(−0.73, 1.83)	0.552(−0.726, 1.830)	1.737(0.484, 6.236)	0.717	0.397
**CAI total score**					
Time*Time					
T1*T0	14.15(10.86, 17.45)	14.153*** (10.857, 17.448)	1400908.593 (51902.128, 37812416.90)	70.847	<0.001
T2*T0	22.29(18.85, 25.74)	22.295*** (10.857, 17.448)	4818724004 (153670727.3, 1.508 + 11)	160.942	<0.001
T2*T1	8.14(4.46, 11.83)				
Group*Time					
Group*T2	27.05(22.42, 31.69)	27.052*** (22.415, 31.689)	5.605E + 11 (5429317329, 5.786 + 13)	130.744	<0.001
Group*T1	15.69 (10.94, 20.43)	15.688*** (10.942, 20.433)	6501217.337 (56476.164, 748383452.4)	41.972	<0.001

*p < 0.05,

**p < 0.01,

***p < 0.001 (two-tailed tests; significance symbols refer to the β coefficients).

### 3.4 Outcomes visualization

**FIGURE 3:**
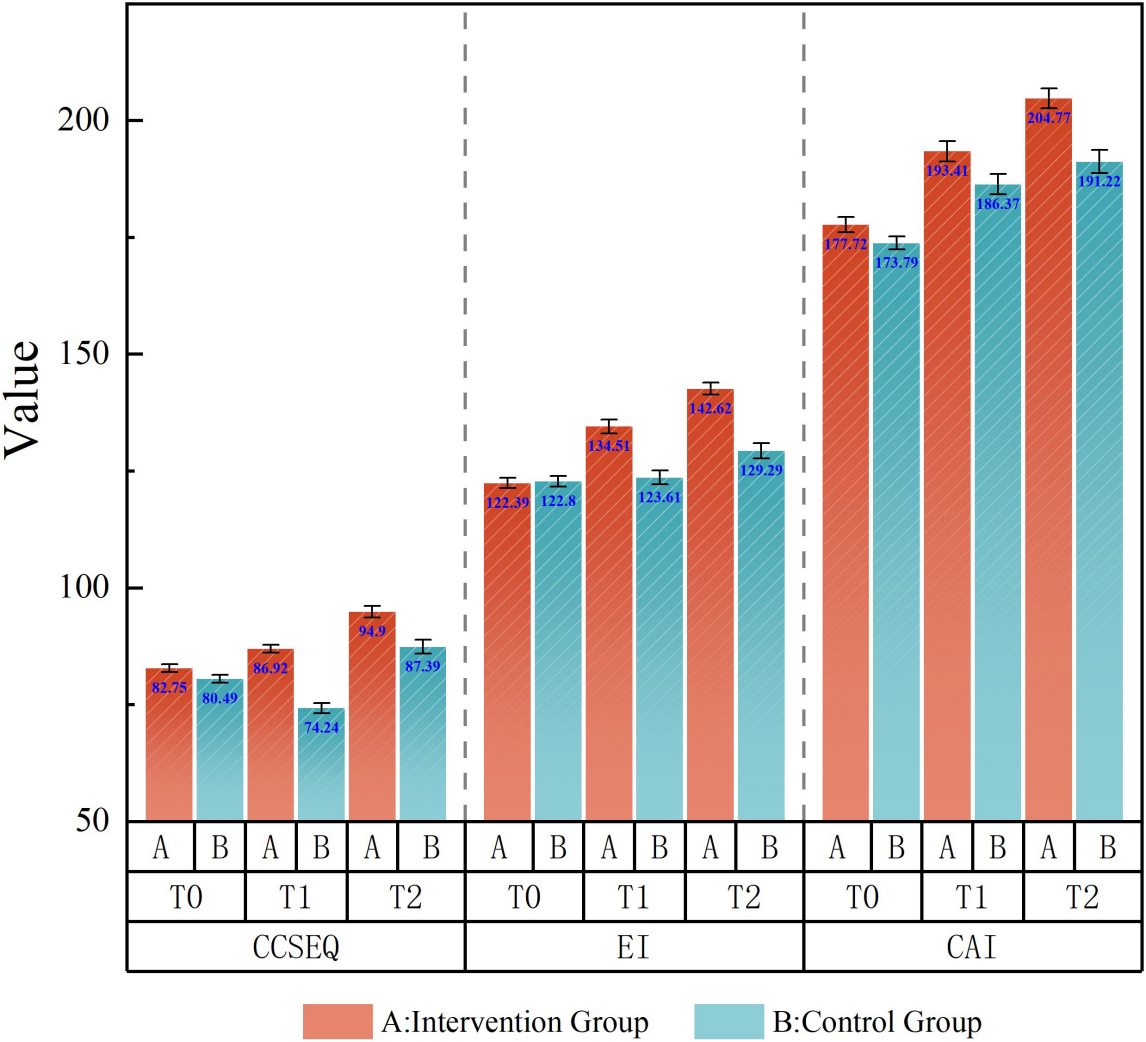
Grouped visualization: the effectiveness of the SCOPE program compared to Standard program overtime.

Figure 3 presents a consolidated visualization comparing the effectiveness of the SCOPE program against the Standard program across the total scores among three scales. This grouped analysis reveals distinct patterns of divergence between the two pedagogical approaches. The SCOPE program demonstrates consistently superior outcomes in all three total scores, indicating holistic enhancement in geriatric nursing competencies. The total CCSEQ score and EIS score both showed steep upward trajectories for the intervention group, while the control group exhibited either minimal improvement or fluctuations. Similarly, the CAI total score steadily increased for the intervention group and only modestly improved in the control group. These graphical trends visually support the statistical findings, highlighting both the immediate and sustained impact of the SCOPE intervention on nursing students’ empathy, emotional intelligence, and caring behaviors.

## 4 Discussion

### 4.1 Comparative impact of the SCOPE and the standard program on nursing competencies

The differing outcomes between the SCOPE program and the Standard Community-based Education program underscored contrasts in pedagogical philosophy and curriculum design. The SCOPE program produced immediate and sustained improvements in empathy and caring behavior. However, the temporary gains in emotional intelligence (EI) raise questions about the long-term impact of competency-based training. In contrast, the control group showed modest, less durable improvements, revealing the limitations of skill-centric nursing education. Nonetheless, the control group’s exposure to clinical environments still contributed to incremental growth, suggesting that experiential learning, while limited, remains valuable. The positive impact of the SCOPE program on empathy, emotional intelligence, and caring behavior is consistent with the broader trend in nursing education that experiential learning is more effective than traditional didactic methods. Prior research has consistently advocated for the integration of community-based experiences into nursing curricula (49). This study provides powerful support for these calls, demonstrating that a structured program combining community engagement, reflection, and skills application leads to significant and sustained improvements. This reinforcement of existing knowledge strengthens the argument for policy changes and curriculum reforms that prioritize experiential learning over passive instruction (50). Furthermore, the finding that caring behavior showed the most substantial gains at the 3-month follow-up (T2) aligns with theories suggesting that professional attributes require extended reinforcement and practice for full integration into a nurse’s repertoire (51).

### 4.2 Theoretical foundations and curriculum design

The SCOPE curriculum aligns with constructivist learning theory, where repeated, scenario-based simulations help students internalize caring behaviors (52). In contrast, the standard program reflects a behaviorist approach, emphasizing procedural mastery rather than psychosocial development. The drop in EI scores at T2 in the SCOPE group suggests that emotional regulation may require more sustained or repetitive exposure. According to Salavera et al. (53), EI exhibits trait like stability, and short-term interventions may be insufficient to shift deeply ingrained emotional traits, especially under post-graduation stress.

Critics argue that simulated settings create artificial separations between training and real world care (54, 55). Although simulations enhance clinical decision making and safety (42), they may oversimplify the emotional and cognitive complexities of geriatric care. Standardized patients cannot replicate cognitive decline or emotional resistance, which may potentially inflate empathy assessments in controlled settings. These concerns mirror critiques by Lin et al. (56) regarding the limitations of decontextualized skills training.

A significant contribution of this research is that it addresses a critical gap: the lack of empirical data on scalable, theoretically informed, and community-based interventions. While many studies on empathy exist, they often fail to provide a replicable model for comprehensive implementation (57). The SCOPE program, with its clear structure, objectives, and sustainable effects, offers a concrete solution. This moves the conversation beyond theoretical appeals for empathy development and provides evidence for a practical and policy related educational strategy.

### 4.3 Sustained gains in empathy and caring behavior

The intervention group’s long-term empathy gains support the neuroplasticity hypothesis, suggesting that repeated interaction with vulnerable populations may strengthen affective pathways (58). In contrast, the control group’s minimal progress in empathy highlights ethical concerns about relying solely on incidental exposure in clinical rotations. Gholamzadeh et al. (52) found that empathy training incorporating reflection and role-playing is more effective than unguided clinical experiences. This supports critiques of the hidden curriculum in nursing, where institutional priorities may inadvertently discourage compassion (59).

The control group showed no significant improvement in the “courage” dimension (CAI scale) or in “understanding others’ emotions” (EIS), exposing a critical gap in traditional training. Standard programs often lack content addressing moral distress or ethical decision-making. In contrast, SCOPE’s scenario-based simulations presented students with dilemmas involving autonomy and family conflict, potentially enhancing their moral reasoning. However, this benefit could be short-lived without ongoing mentorship, risking the perception of courage as a checklist item instead of an internalized value.

Further analysis of the control group confirms these shortcomings. The lack of significant gains between T1 and T2 in the courage dimension of caring behavior (in CAI) (*p* = 0.128; *p* = 0.086; *p* = 0.750) aligns with prior findings by Gholamzadeh et al. (52). Traditional training failed to induce meaningful emotional or behavioral change, although it may still help develop technical skills, particularly in low-resource settings lacking simulation infrastructure. On the other hand, Bauchat et al. (54) argue that caring behavior relies heavily on practical communication skills and that simulation training provides the opportunity to improve those skills. Bearman et al. (55) confirm that students make fewer mistakes and improve safety in actual patient care by identifying and correcting errors in a simulation environment. It was also confirmed that simulation training allows students to receive immediate feedback and self-assessment after performing caring behavior, which is critical for continuous improvement.

### 4.4 Emotional intelligence: gains and limits

SCOPE participants showed initial improvements in EI, but these gains regressed at follow-up. This aligns with Salavera et al. (53), who argued that emotional intelligence, while somewhat malleable, requires ongoing reinforcement. Zeidner et al. (60) further contend that EI development stagnates without structural support. In hierarchical hospital settings, expressing emotional vulnerability may be discouraged, reducing the practical value of EI training. This underscores the disconnect between individual level interventions and institutional norms.

The control group’s minimal improvement in EI challenges the belief that clinical exposure naturally builds emotional competence. Lönn et al. (61) warns that unsupervised patient interaction may encourage emotional detachment rather than empathy, particularly in high-stress fields like geriatrics. SCOPE’s guided debriefings may buffer students against this emotional numbing by creating space for reflection.

### 4.5 Simulation and caring behavior

Caring behavior was the most distinct domain between the two programs. The SCOPE group benefited from structured simulations that encouraged practice and feedback, helping students internalize caring acts. This aligns with Bauchat et al. (54) and Bearman et al. (55), who found that simulation fosters clinical empathy and reduces errors. In contrast, the standard program assumed caring would emerge organically through practical notion that lacks empirical support. Cho and Kim (42) emphasize that caring must be developed through deliberate, repeated exposure.

The elderly people’s feelings of stiff joints, blurred vision, hearing loss and back pain were given to the nursing students to experience the physiological decline of the older people, which had already been tested in previous studies, that help with their cognitive development (62, 63). The nursing students reflected that they felt helpless and hurt physically when they got a stiff back and how hard it was to climb stairs when they stepped into the older people’s shoes, the same feeling described by Weekes and Phillips (64). Nevertheless, simulations have limitations. They may fail to capture the emotional unpredictability of real-life care settings (65). Over-reliance on simulations could lead to unrealistic expectations of clinical interactions, where outcomes are not always predictable or structured.

### 4.6 Toward an integrated nursing curriculum

The findings suggest that an integrated curriculum could maximize the strengths of both approaches. SCOPE’s structured empathy and caring exercises could be embedded within community rotations to blend simulation with real-life complexity. However, this integration requires trained faculty and resources, which may not be feasible in all settings. Nursing students watch the caring behavior of the teacher in action and perform simulated experiments. As the study revealed, the emotions evoked by personal care activities could significantly improve their attitude toward older people and trigger their empathetic emotions (66). At the same time, their confidence was also supported when they focused on the patients’ needs (67). Referring to the scenarios based on the older peoples’ life in community, these critical life situations impact one’s emotional control and can trigger stress (68). Nevertheless, this is one of the styles of the cognitive transactional model of stress-appraisal-coping, which has been indicated in nursing students’ clinical placement process to make sense of life (69). It has been proved that the role of simulation in nursing education is well-established for facilitating skill acquisition, critical thinking, and confidence in a controlled environment, which is also aligns with the research of Alanazi et al. (70).

Importantly, neither this study nor that of Gholamzadeh et al. (52) included long-term follow-up beyond T2. Without this, it remains unclear whether brief interventions can foster enduring professional identity or only temporary behavioral change. As nursing education shifts toward competency-based models, future research must explore how to sustain affective learning over time.

### 4.7 Strengths and limitations

This study has several strengths. The SCOPE program adopted a well-rounded approach that combined classroom learning, simulation, and hands-on community experience, offering nursing students both theoretical knowledge and practical skills. It focused on developing empathy, emotional intelligence, and caring behavior-key components of high-quality geriatric care. Data was collected at multiple points, helping to observe changes over time. Involving both campus and community settings also increased the practical relevance of the findings.

However, there are some limitations. The 4-week intervention period was relatively short, which may not have been enough to create lasting changes. Limited opportunities for real-life application in community settings may have reduced the depth of learning. Since the study was conducted within a specific cultural and educational setting, the findings may not be easily applied elsewhere.

Future research should explore ways to integrate community-based programs like SCOPE more deeply into nursing curricula, either as dedicated modules or through existing courses, while addressing barriers such as limited faculty resources and time constraints. Tailoring educational strategies based on students’ backgrounds and incorporating tools like reflective practices or digital feedback platforms could further enhance empathy and emotional intelligence development. Methodologically, future studies would benefit from randomisation and qualitative approaches to provide more robust and well-rounded insights.

## 5 Conclusion

While the SCOPE program outperforms standard education in cultivating empathy and caring behavior, its design limitations-episodic EI training, simulation-reality disparities—reveal broader tensions in competency-based nursing education. Future iterations should incorporate longitudinal EI reinforcement mechanisms and workplace culture alignment, while standard programs must integrate deliberate empathy training to counterbalance their operational focus. Ultimately, nurturing humanistic nurses requires transcending the false dichotomy between technical proficiency and psychosocial intelligence-a goal demanding curricular innovation and systemic reform in equal measure. Ultimately, while the SCOPE program proves advantageous in shaping psychosocial competencies, the results also reveal areas needing refinement, particularly in sustaining gains in emotional intelligence over time. One explanation may be the one-time nature of the intervention, lacking longitudinal reinforcement. Another concern lies in transferability: although effective in this controlled trial, the program’s scalability to different cultural and institutional settings remains uncertain. As such, while the results of this study reject the null hypothesis (H01), confirming the SCOPE program’s significant effects at T0, T1, and T2, they also call for a cautious interpretation. Future research should explore hybrid models that combine the strengths of both approaches-pairing technical skills training with affective education-to cultivate both competence and compassion in future nurses.

## Data Availability

The original data supporting the conclusions of this article are available from the corresponding author upon reasonable request.
